# Network pharmacology combined with experimental validation show that apigenin as the active ingredient of *Campsis grandiflora* flower against Parkinson’s disease by inhibiting the PI3K/AKT/NF-κB pathway

**DOI:** 10.1371/journal.pone.0311824

**Published:** 2024-10-09

**Authors:** Kai Liu, Jing An, Jing Zhang, Jihu Zhao, Peng Sun, Zhaohui He

**Affiliations:** 1 Department of Neurosurgery, The First Affiliated Hospital of Chongqing Medical University, Chongqing, China; 2 Department of Neurosurgery, People’s Hospital of Rizhao, Jining Medical College, Rizhao, Shandong, China; 3 Department of Pathology, People’s Hospital of Rizhao, Jining Medical College, Rizhao, Shandong, China; 4 Department of Pharmacy, Jining Medical College, Rizhao, Shandong, China; 5 Department of Neurosurgery, The Affiliated Hospital of Qingdao University, Qingdao, China; The Second Affiliated Hospital of Guangzhou Medical University, CHINA

## Abstract

The exploration of novel natural products for Parkinson’s disease (PD) is a focus of current research, as there are no definitive drugs to cure or stop the disease. *Campsis grandiflora* (Thunb.) K. Schum (Lingxiaohua) is a traditional Chinese medicine (TCM), and the exact active constituents and putative mechanisms for treating PD are unknown. Through data mining and network pharmacology, apigenin (APi) was identified as the main active ingredient of Lingxiaohua, and key targets (TNF, AKT1, INS, TP53, CASP3, JUN, BCL2, MMP9, FOS, and HIF1A) of Lingxiaohua for the treatment of PD were discovered. The primary routes implicated were identified as PI3K/AKT, Apoptosis, TNF, and NF-κB pathways. Subsequently, therapeutic potential of APi in PD and its underlying mechanism were experimentally evaluated. APi suppressed the release of mediators of inflammation and initiation of NF-κB pathways in MES23.5 cells induced by MPP^+^. APi suppressed caspase-3 activity and apoptosis and elevated p-AKT levels in MES23.5 cells. Pretreatment with LY294002, a PI3K inhibitor, resulted in APi treatment blocking the activation of NF-κB pathway and expression of inflammatory factors in MES23.5 cells by activating the PI3K/AKT pathway. In conclusion, APi protects dopaminergic neurons by controlling the PI3K/AKT/NF-κB pathway, giving novel insights into the pharmacological mechanism of Lingxiaohua in treating PD.

## Introduction

Worldwide, the incidence of Parkinson’s disease (PD) is increasing with the aging of the population, placing a heavy burden on society and the economy [1, 2]. One of the key factors influencing the progression of PD is neuroinflammation, which leads to dopaminergic neuronal damage through oxidative stress and apoptosis [3, 4]. Due to the incurability of PD, the pharmaceutical industry faces some tough challenges, especially increased outlays and unsustainable risk management. There is a major change in drug research from synthetic moieties to plant-based formulations [5]. Natural products of plant source have been used for many years to treat neurological disorders. However, the molecular pathways by which natural compounds protect against PD are unclear.

*Campsis grandiflora* (Thunb.) K. Schum is a climbing plant cultivated in China, Korea and Japan. It is known as "Lingxiaohua" in Chinese and is used to treat disorders associated with blood stasis [6, 7]. Modern research has confirmed the presence of valuable compounds Lingxiaohua in the form of triterpenoids, flavonoids, phenolic acids, and volatile oils [8, 9]. Meanwhile, large amounts of research suggests that plant extracts containing triterpenes or flavonoids have various beneficial effects in PD owing to their anti-inflammatory and anti-oxidation functions [10–14]. The 50% ethanol (EtOH) extract of Lingxiaohua showed anti-oxidation and anti-inflammatory potential in vitro, both of which are hallmarks of neurodegenerative diseases [9]. Yu et al. applied 50% EtOH to extract Lingxiaohua and the extract obtained was further partitioned by ethyl acetate (EtOAc). The results revealed that the EtOAc extract of Lingxiaohua has antioxidant and antidepressant-like properties [8]. Neurodegenerative diseases, especially PD, are known to have a high comorbidity with a wide range of mental health conditions [15, 16]. Tizabi et al. proposed that factors that prevent depressive disorders may also prevent PD from developing and/or progressing [17]. No toxicity has been found for the Lingxiaohua, which is usually consumed as a medicinal meal [6, 18]. Thus, Lingxiaohua could potentially serve as a drug or dietary supplement in managing PD.

Based on existing studies, many natural products based on the phosphatidylinositol 3-kinase/protein kinase B (PI3K/AKT) pathway may protect dopamine (DA) neurons and thus play a role in the prevention and treatment of PD [19]. AKT has three highly homologous subtypes. Among them, AKT1 plays multiple roles in regulating cell survival, differentiation and proliferation [19, 20]. Its activated state is strongly linked to PI3K. The PI3K inhibitor LY294002 is also able to block AKT1 activation [21, 22]. Low expression of phospho-AKT (p-AKT) was found in DA neurons of postmortem Parkinson’s patients, suggesting that disruption of PI3K/AKT signals is linked to the pathophysiology of PD [23, 24]. AKT1 is also considered a key regulator of the neuroinflammatory response. It exhibits a therapeutic potential in PD models by mediating NF-κB pathway [24, 25]. Nuclear translocation of NF-κB is critical for several inflammatory cytokines, such as TNF-α, G-CSF, IL-6, etc., which induce dopaminergic neurons death [26, 27].

To analyze the molecular mechanism of apoptosis in PD cells, we established a cell model using MPP^+^-induced MES23.5 cell injury for validation. As a hybrid cell, MES23.5 dopaminergic cell can exhibit some similar properties to primary neurons derived from substantia nigra (SN) [28]. Multiple pathways have been implicated in MPP^+^-mediated neurotoxicity in MES23.5 cells, such as the triggering of oxidative stress [29], promotion of apoptosis [30], and inactivation of the PI3K/AKT cascade [31]. Network pharmacology can reflect the pathways of effect of herbal medicines in complex disease networks, which can help predict the potential mechanisms of drug therapy for PD, thus supporting the discovery of potential natural products for the therapy of PD [32, 33]. Here, network pharmacology was used to investigate Lingxiaohua’s compounds, targets and pathways for PD. Subsequently, the role of apigenin (APi), the main bioactive component of Lingxiaohua, was investigated in a PD cell model to provide important theoretical support for in-depth exploration of Lingxiaohua in treating PD.

## Materials and methods

### Reagents

APi was obtained by Meilun Biotechnology (Dalian, China). LY294002 (an inhibitor of PI3K) and antibodies against NF-κB p65, p-NF-κB p65, AKT, p-AKT were supplied by Cell Signaling Technology, Inc. (MA, USA). Antibody against PCNA and β-actin was supplied by Santa Cruz Biotechnology, Inc. (CA, USA). Inflammatory factor ELISA kits were purchased from Biolegend (CA, USA). Local commercial sources supplied all other chemicals and reagents.

### Retrieving common targets of Lingxiaohua and PD

Traditional Chinese medicine Systems Pharmacology (TCMSP, https://tcmspw.com/tcmsp.php) database was used to search for the chemical composition of Lingxiaohua. In order to screen drug active ingredients, the oral bioavailability (OB) set value should be ≥ 20% and drug likeness (DL) set value should be ≥ 0.18. A search for PD-related targets was performed in four databases: GeneCards (https://www.genecards.org/), OMIM (https://omim.org/), DrugBank (https://go.drugbank.com/), and PharmGKB (https://www.pharmgkb.org/). The above searched targets were de-duplicated and merged into a disease-related target dataset. The STRING database (https://string-db.org/) was performed to construct of Protein-Protein Interation (PPI) network of overlapping targets. The high confidence higher than 0.4 that was the lowest interaction threshold to generate PPI network plot. The Degree value, Between Centrality (BC) and Closeness Centrality (CC) of each node in the PPI network were counted and analyzed.

### Ingredient-Disease-Target-Pathway network establishment

Gene Ontology (GO) and Kyoto Encyclopedia of Genes and Genomes (KEGG) enrichment analysis of potential targets for PD drug prevention and treatment were accomplished using Metascape database (https://metascape.org/gp/index.html) and visualized with Microbiology online mapping platform (http://www.bioinformatics.com.cn/) to demonstrate the role of screened targets. The intersecting targets, Lingxiaohua active ingredients and KEGG pathways were organized in Excel according to the data files and attribute files, and imported into Cytoscape 3.10.1 software to eatablish Ingredient-Target-Pathway (I-T-P) network. Its built-in network analyzer analyzes network topology parameters and identifies the most important active components.

### Cell viability assay

MES23.5 cells were obtained and cultured as previously described [34]. The 3-(4,5-dimethylthiazol-2-yl)-2,5-diphenyltetrazolium bromide (MTT) is a test for cell survival and growth. In this experiment, MES23.5 cells were pretreated with APi (5, 10, and 15 μmol/L) for 2 h, and then cocultured with MPP^+^ (200 μmol/L) and APi for another 24 h. After incubation, cell viability was measured using a colorimetric assay (TECAN, Austria).

### ELISA experiments

MES23.5 cells were seed into polylysine-coated sterile 6-well plates and pretreated with LY294002 (5 μM) for 1 h or APi (10 μmol/L) for 2h, and then exposured to MPP^+^ (200 μmol/L) for specific time. After processing of MES23.5 cells, levels of inflammatory factors such as TNF-α were evaluated according to the Instruction of the ELISA kit.

### Detection of cell apoptosis

MES23.5 cells were first treated with APi (10 μmol/L) for 2 h and then stimulated with MPP^+^ (200 μmol/L) for 24 h, and apoptosis assay was performed. Caspase-3 (CASP3) activity was detected through a PE-coupled monoclonal active caspase-3 antibody apoptosis kit (BD Biosciences Pharmingen, USA) according to a method previously described by our group [34]. Briefly, cells were treated as said by the kit instructions and examined by flow cytometry (Becton Dickinsion, USA) under 523 nm excitation and 658 nm emission. Similarly, we used Hoechst 33258 staining to detect apoptotic nuclei. MES23.5 cells were secured in 4% paraformaldehyde for 10 minutes and then discolored with Hoechst 33258 dye consistent with instructions (Beyotime, China). Using a fluorescence microscope (Zeiss, Germany) to observe and photograph the results. The ratio of apoptotic nuclei to normal nuclei was observed in 10 randomly selected fields of view for each sample.

### Immunofluorescence staining

Nuclear translocation of NF-κB p65 subunit was performed to detect by immunofluorescence staining. MES23.5 cells were dealed with APi (10 μmol/L) for 2h and MPP^+^ (200 μmol/L) for another 2h. Followed by incubating overnight at a 1:500 dilution with anti-NF-κB p65 antibody. After PBS washing, fluorescence labeling was performed using secondary antibodies labeled with Alexa Fluor 555 (1:500). The steps following previously described [35].

### Western blot analysis

As previously described [36], nuclear and cytoplasmic proteins were extracted using the corresponding protein extraction kits, respectively (Beyotime, Jiangsu, China). MES23.5 cells were lysed as stated by the lysis buffer instructions, and protein concentrations were identified prior to separation by SDS-PAGE. Membranes were incubated with AKT (1:1,000), p-AKT (1:1,000), NF-κB p65 (1:2,000), p-NF-κB p65 (1:2,000) antibodies. As a loading control for total tissue lysate, β-actin (1:10,000) was used. For nuclear samples, PCNA (1:1,000) was used as a nuclear loading control.

### Statistical analysis

Data were presented as mean ± S.E.M. and multi-group comparisons were performed using one-way ANOVA and Student-Newman-Keuls test. *P* < 0.05 was believed statistically significant.

## Results

### Retrieving common targets of Lingxiaohua and PD

**Fig 1:**
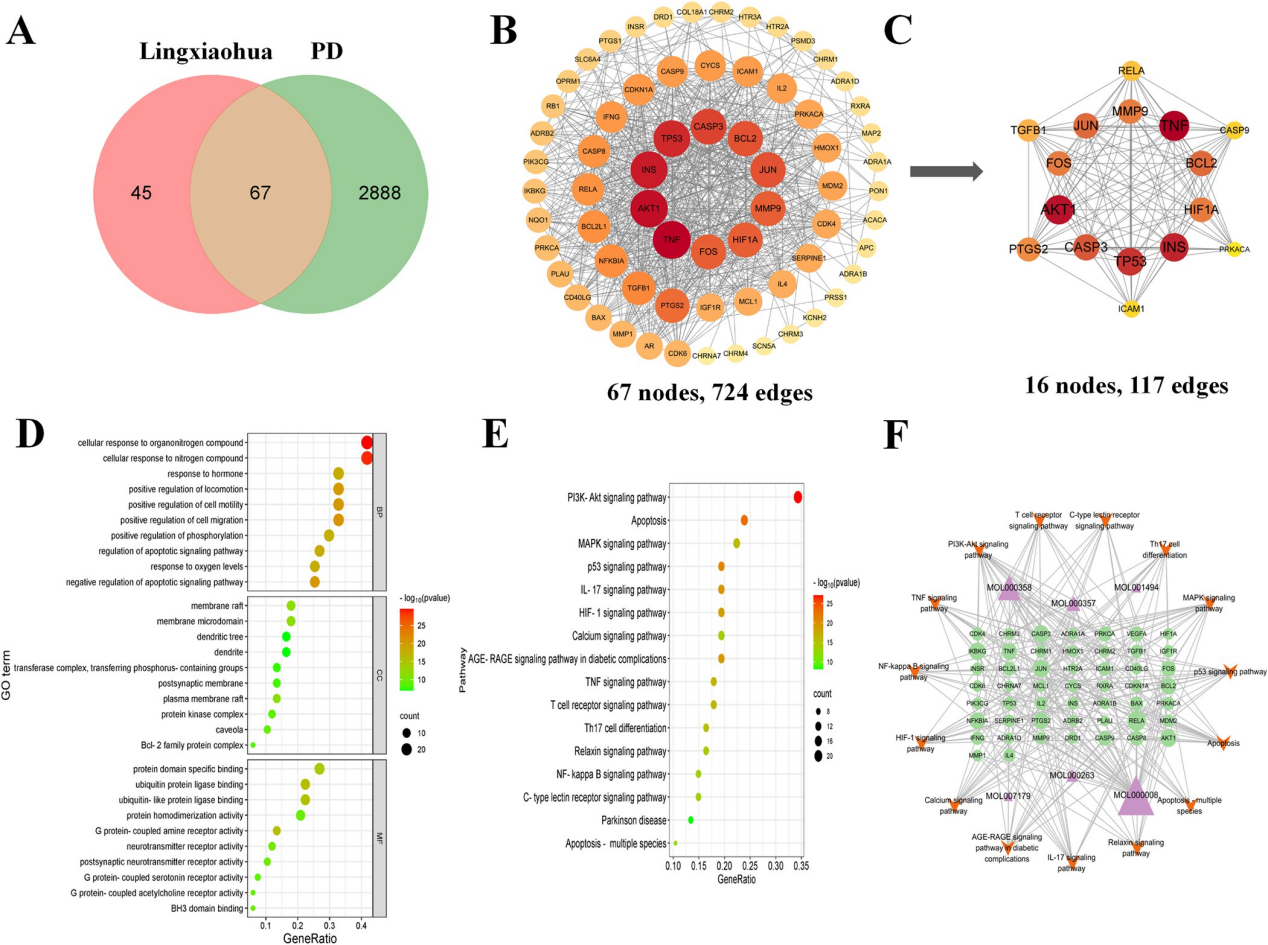
Network pharmacology predicted possible targets, pathways, and active ingredients for the anti-PD effects of Lingxiaohua. (A) Common targets of Lingxiaohua and PD. (B) PPI network of putative targets. (C) PPI core network. (D) GO analysis. (E) The top 15 KEGG pathways and PD signaling pathways. (F) I-T-P network establishment. Purple triangles indicate components of Lingxiaohua, green ovals indicate common goals, and orange inverted triangles indicate major pathways. The size of a node is proportional to its degree. MOL000008, apigenin; MOL000358, beta-sitosterol; MOL000263, oleanolic acid; MOL000357, sitogluside; MOL001494, Mandenol; MOL007179, Linolenic acid ethyl ester.

The TCMSP database searched for 10 pharmaceutical ingredients of Lingxiaohua (**S1 Table**). Combined with Uniport to normalize the drug targeting, a cluster of 146 possible drug targets of Lingxiaohua were predicted, and conserved a cluster of 112 drug targets after removing duplicate data. We obtained 2442 targets in GeneCards database, 545 targets in OMIM database, 84 targets in DrugBank database and 69 targets in PharmGKB database. The above targets were combined to create 2995 PD-related targets that met the requirements. Venn diagram showing 67 common targets between Lingxiaohua and PD (**Fig 1A**). A PPI network consisting of 67 nodes and 724 edges was constructed in the String database, and TNF was primary target in the network (Degree = 48, BC = 0.0825, CC = 0.7674) (**Fig 1B**). For clarity, we further extract the PPI core network containing 16 nodes and 117 edges (**Fig 1C**). TNF, AKT1, INS, TP53, CASP3, JUN, BCL2, MMP9, FOS and HIF1A may be the key targets for treating PD by Lingxiaohua.

### Ingredient-Disease-Target-Pathway network establishment

To gain a further underlying mechanism of action of Lingxiaohua for PD, an enrichment analysis was performed on 67 potential targets on the Metascape platform. As shown in **Fig 1D**, the majority of biological processes (BP) were related to cellular responses to organonitrogen compounds, regulation of apoptotic signalling pathways, regulation of phosphorylation, and so forth. The majority of cell components (CC) were related to membrane rafts, membrane microdomain, and so forth. Finally, the majority of molecular functions (MF) were related to, among other things, protein domain specific binding. KEGG enrichment analysis revealed 174 pathways involved. Top 15 KEGG pathways and PD signalling in vivo pathomechanisms with the lowest *P*-value after excluding other disease pathways are shown in **Fig 1E**. The studies focused primarily on PI3K/AKT, apoptosis, TNF and NF-κB pathways. We obtained the six main components of Lingxiaohua for PD from the I-T-P network, which were apigenin, beta-sitosterol, sitogluside, oleanolic acid, mandenol and linolenic acid ethyl ester. Among them, apigenin was the most significant component (Degree = 37, BC = 0.3122, CC = 0.5966), followed by beta-sitosterol (Degree = 19, BC = 0.1763, CC = 0.4522). Furthermore, the 5 most prominent targets in the network were AKT1, BCL2, RELA, CASP3, and NFKBIA (**Fig 1F**).

### APi enhanced cell survival and attenuated inflammatory responses

**Fig 2:**
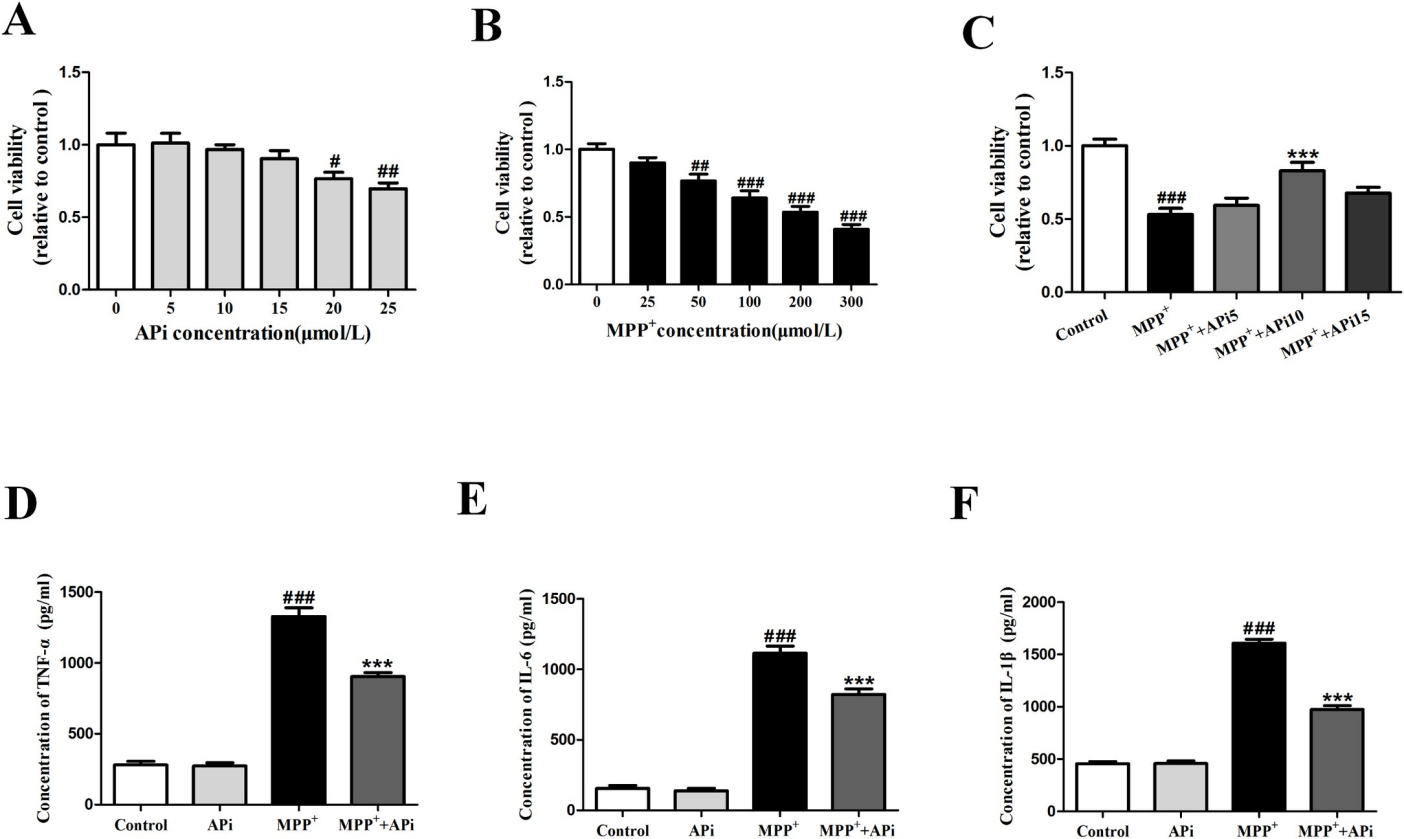
APi enhanced cell survival and attenuated inflammatory responses. (A) Cell viability of MES23.5 cells exposed to APi for 26 h. (B) Cell viability of MES23.5 cells exposed to MPP^+^ for 24 h. (C) Cell viability assay of MES23.5 cells after pretreatment with APi (5, 10, 15 μmol/L) for 2 h and then co-treatment with MPP^+^ (200μmol/L) for 24 h. Then, TNF-α (D), IL-6 (E) and IL-1β (F) levels were detected by ELISA. Data were expressed as mean ± S.E.M., n = 6 (^#^*P* < 0.05, ^##^*P* < 0.01, ^###^*P* < 0.001 compared with control group; ***P < 0.001 compared with MPP^+^ group).

When the concentration of APi was greater than or equal to 20 μmol/L, the results showed that the viability of MES23.5 cells decreased. Treatment of cells with 200 μmol/L MPP^+^ resulted in a 46.43% decrease in cell viability. MTT assay demonstrated that 10 μmol/L APi antagonized the decrease in cell viability induced by MPP^+^ (**Fig 2A–2C**). Consequently, the concentration of 10 μmol/L APi was employed in the subsequent experiments. In the inflammatory response assay, inflammatory factors content was tested by ELISA. APi was identified as an inhibitor of inflammatory factors production, suggesting that APi inhibits inflammatory response in MPP^+^-activated MES23.5 cells (**Fig 2D–2F**).

### APi inhibited MPP^+^-induced translocation of NF-κB p65 subunits

**Fig 3:**
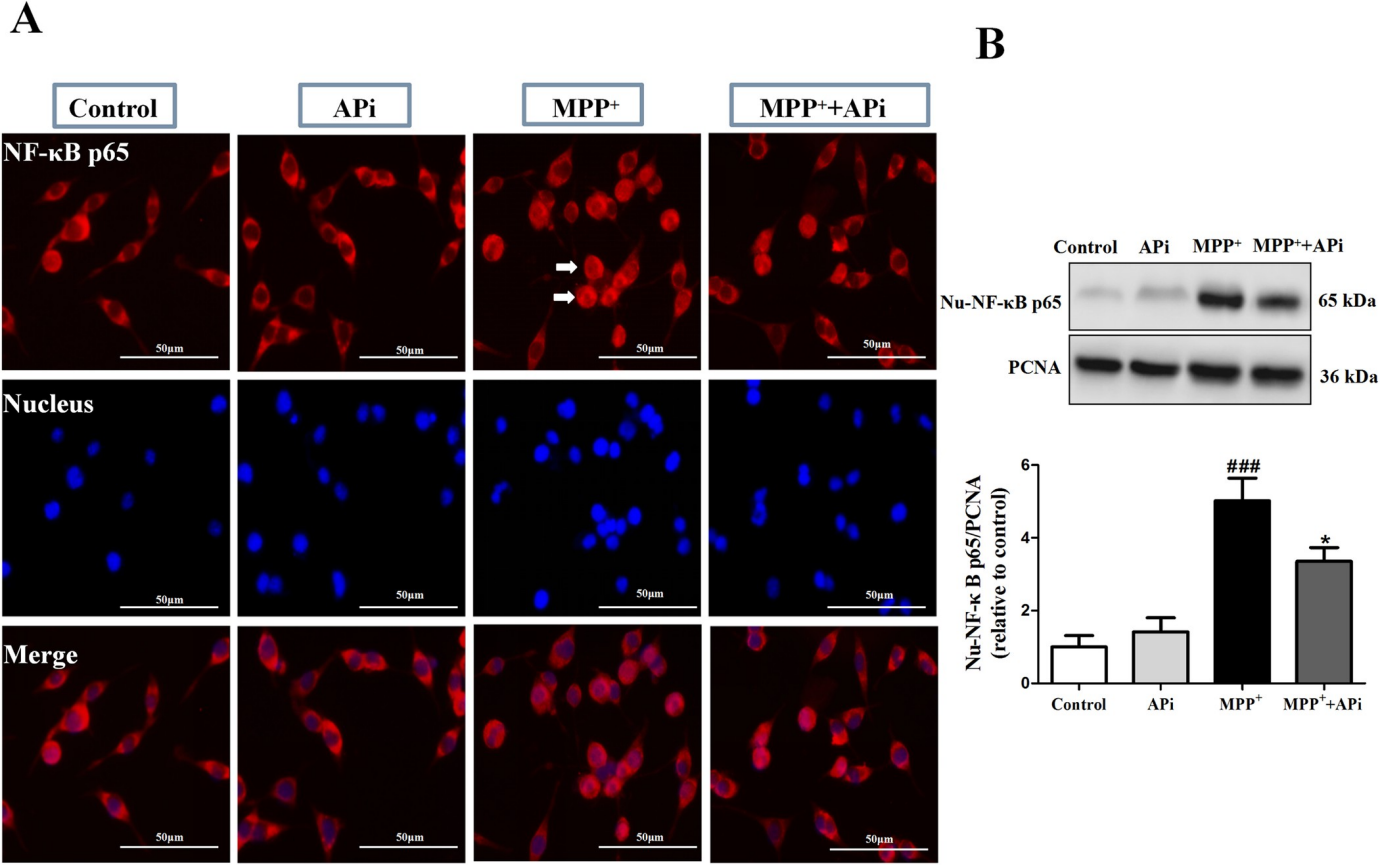
APi inhibited MPP^+^-induced translocation of NF-κB p65 subunits. Cells were first treated with APi (10 μmol/L) for 2 h and then stimulated with MPP^+^ (200 μmol/L) for 2 h. (A) Immunofluorescence assay for nuclear translocation of NF-κB p65. Representative images of NF-κB p65 expression (arrowheads). (B) Expression of the intranuclear protein NF-κB p65 was detected by Western blotting. PCNA was used as an internal control for nucleolysis (n = 3). Data were expressed as mean ± S.E.M. (^###^*P* < 0.001 compared with control group; *P < 0.05 compared with MPP^+^ group).

To ascertain whether the impact of APi on neuroinflammation is linked to the NF-κB pathway, we used immunofluorescence and western blotting to detect NF-κB p65 protein levels in cells. The results showed that MPP^+^ could increase the NF-κB p65 protein content in the nucleus of cells, as evidenced by an increase in red fluorescence at the nuclear site of MES23.5 cells as well as an increase in intracellular Nu-NF-κB p65 protein content. The application of APi could ameliorate the above situation (**Fig 3**).

### APi inhibited CASP3 activity in cells and its mediated apoptosis

**Fig 4:**
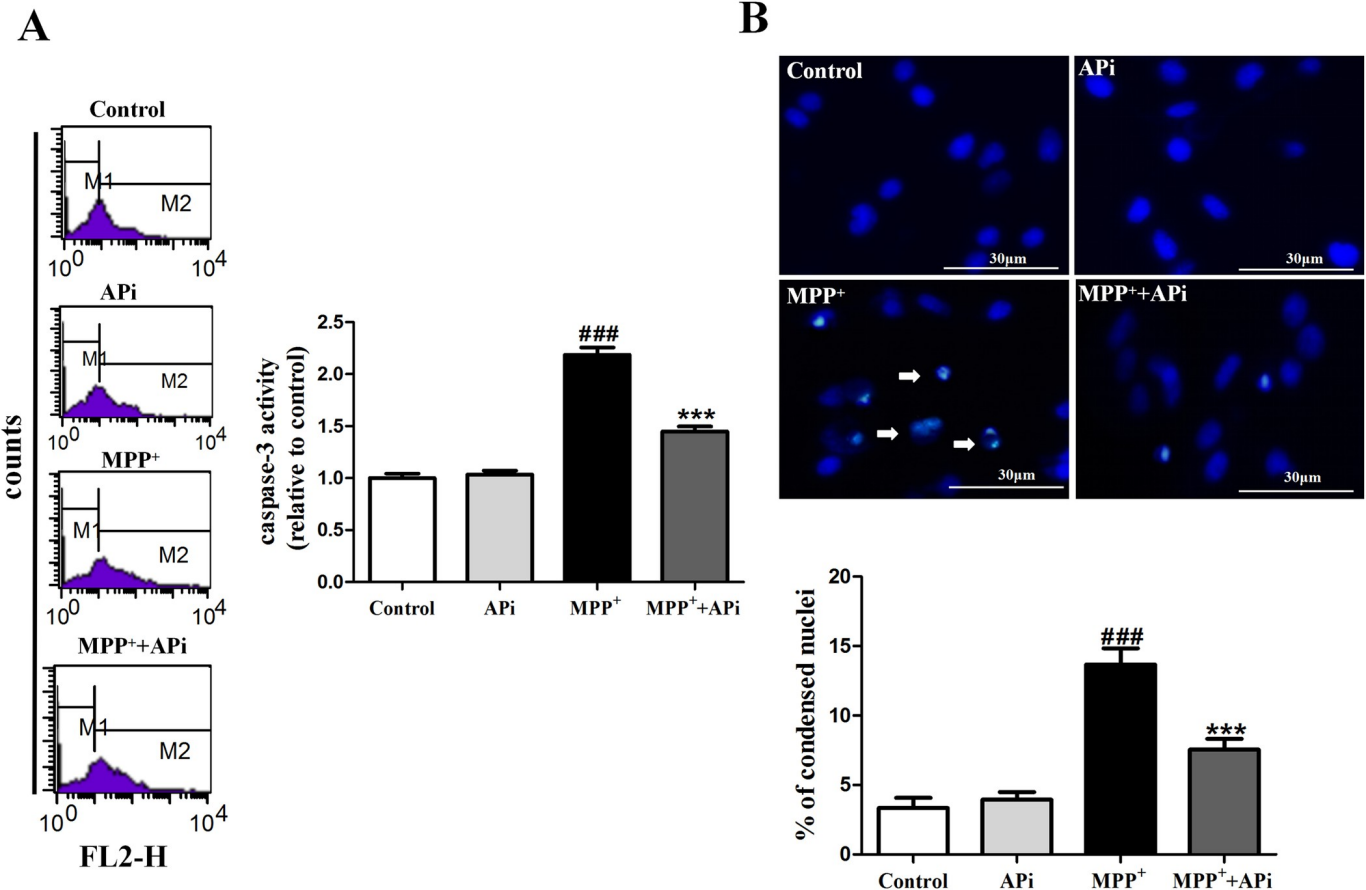
APi inhibited CASP3 activity in cells and its mediated apoptosis. Cells were first treated with APi (10 μmol/L) for 2 h and then stimulated with MPP^+^ (200 μmol/L) for 24 h. (A) Representative graphs and statistical analysis of fluorescence assay of CASP3 activity in different groups (n = 6). (B) Representative graphs and statistical analysis of Hoechst 33258 staining in different groups (n = 10); nuclei of MPP^+^-treated cells appeared hypercondensed (bright staining) and showed chromatin fragmentation (indicated by arrows). Data were expressed as mean ± S.E.M. (^###^*P* < 0.001 compared with control group; ***P < 0.001 compared with MPP^+^ group).

Flow cytometry results showed that APi was able to inhibit the MPP^+^-induced increase in CASP3 activity. Meanwhile, as in the previous results, there was no statistically significant difference between the results of the APi group and the control group (**Fig 4A**). Apoptosis is characterized by nuclear condensation and broken DNA. We used Hoechst 33258 staining to observe the morphological changes of cell nuclei. Nuclear staining showed that MPP^+^ can cause nuclear condensation and APi can attenuate this effect (**Fig 4B**).

### APi activated the PI3K/AKT pathway

**Fig 5:**
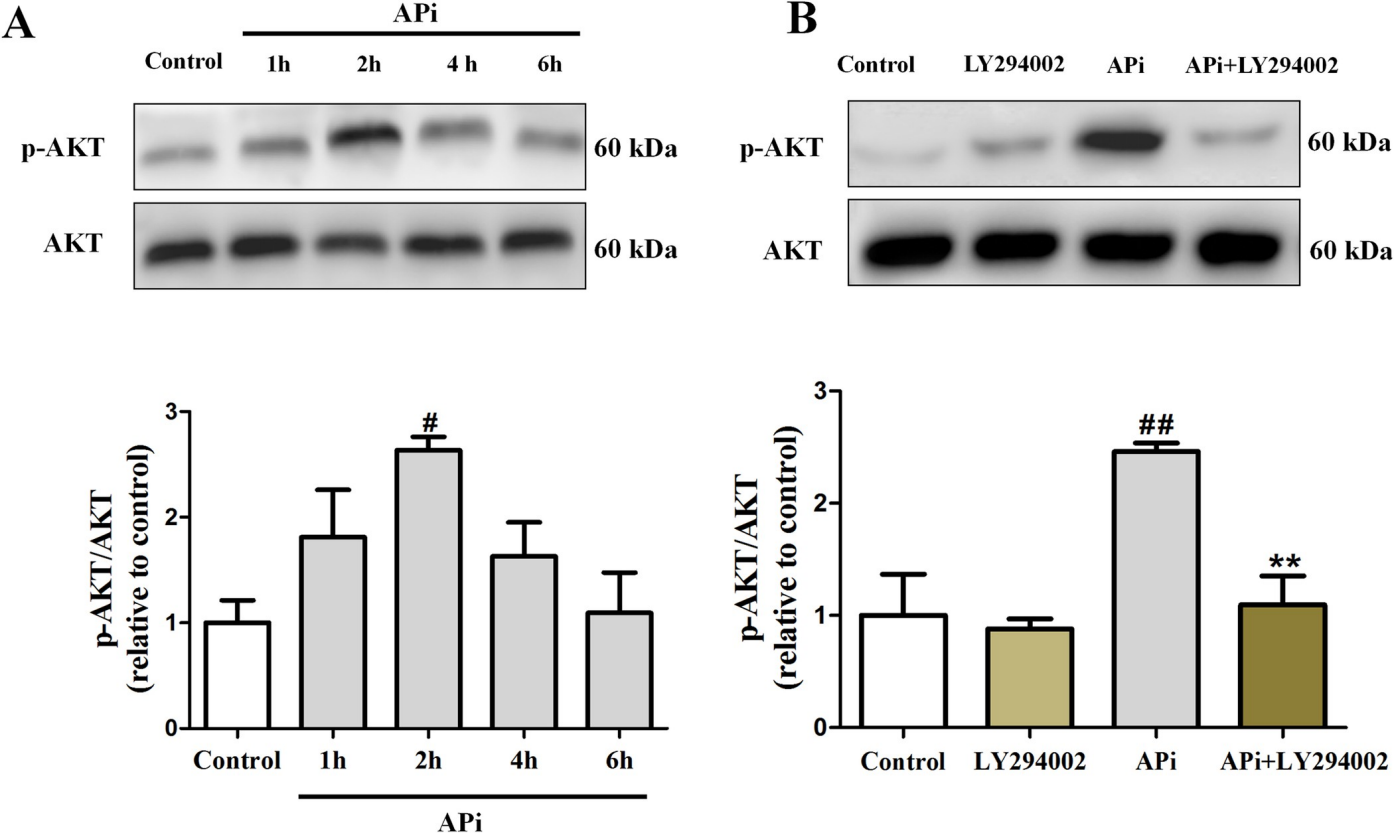
APi activated the PI3K/AKT pathway. (A) Cells were incubated with APi (10 μmol/L) for different times (0, 1, 2, 4, and 6 h) and p-AKT and AKT were measured by Western blotting (n = 3). (B) Cells were pretreated with PI3K inhibitor LY294002 (5 μM) for 1h, following by incubation with APi (10 μmol/L) for 2h. The p-AKT and AKT were then measured by Western blotting (n = 3). Data were expressed as mean ± S.E.M. (^#^*P* < 0.05, ^##^*P* < 0.01 compared with control group; **P < 0.01 compared with APi group).

To determine APi’s direct effect on the PI3K/AKT pathway, we conducted an evaluation of the effect of APi on p-Akt and a comparison of the barrier action of LY294002 (an inhibitor of PI3K) in MES23.5 cells. We found time-dependent changes in p-Akt levels after exposure to APi for varying durations (0, 1, 2, 4, and 6 h), and the maximal effects occurred at 2h (**Fig 5A**). And AKT phosphorylation was effectively blocked by LY294002 (**Fig 5B**).

### APi inhibited NF-κB p65 phosphorylation and inflammatory factors expression through activation of PI3K/AKT pathway

**Fig 6:**
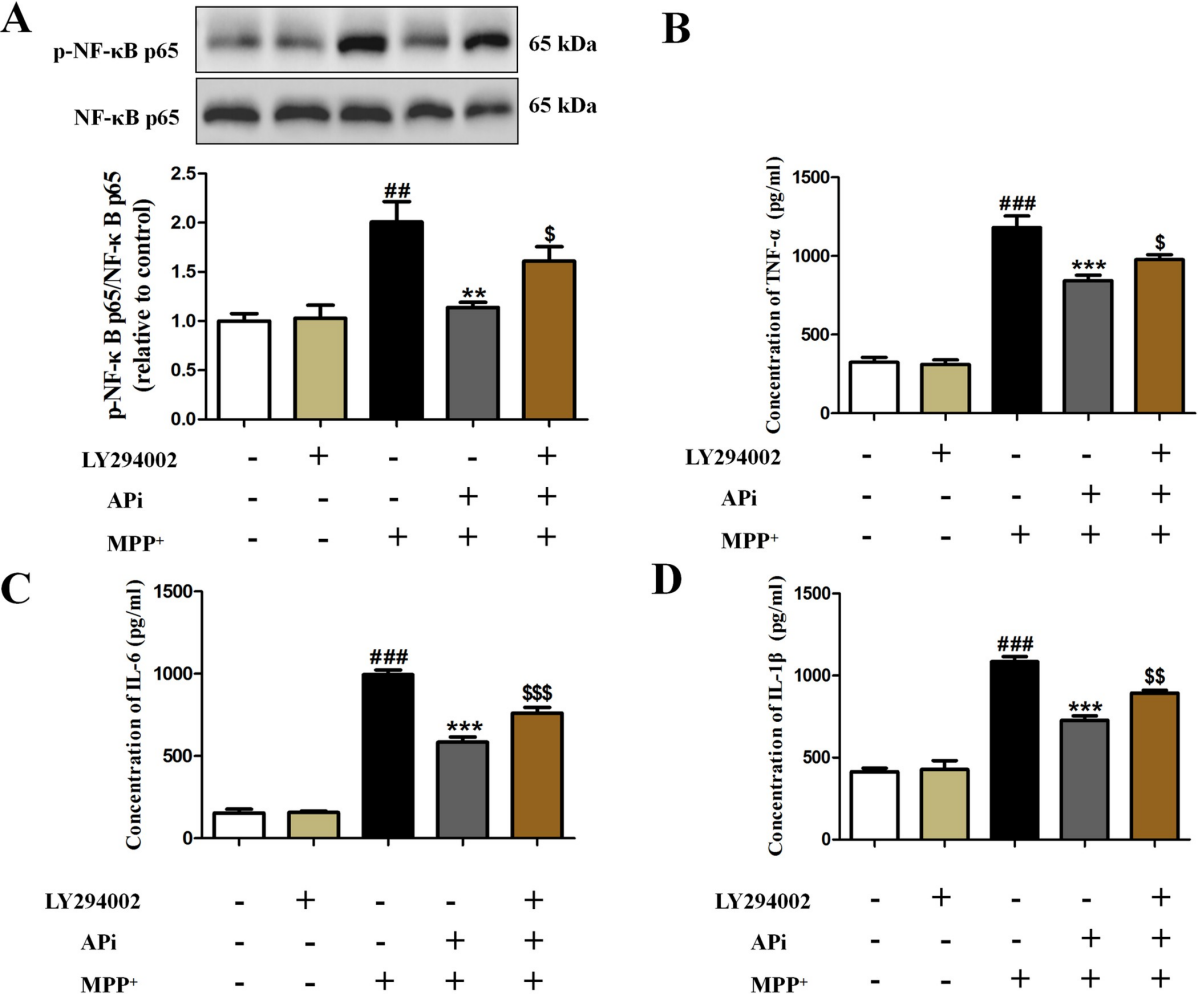
APi inhibited NF-κB p65 phosphorylation and inflammatory factors expression by activating PI3K/AKT pathway. Cells were pretreated with PI3K inhibitor LY294002 (5 μM) for 1h, followed by incubation with APi (10 μmol/L) for 2h, then stimulated with MPP^+^ (200 μmol/L) for 2 h. (A) The p-NF-κB p65 and NF-κB p65 were measured by Western blotting (n = 3). TNF-α (B), IL-6 (C) and IL-1β (D) levels were determined by ELISA (n = 6). Data were expressed as mean ± S.E.M. (^##^*P* < 0.01, ^###^*P* < 0.001 compared with control group; **P < 0.01, ***P < 0.001 compared with MPP^+^ group; ^$^*P* < 0.05, ^$ $^*P* < 0.01, ^$ $ $^*P* < 0.001 compared with MPP^+^+APi group).

In further clarification of the possible relationship between anti-inflammatory efficiency of APi and triggering of the PI3K/AKT cascade, MES23.5 cells were pre-treated with LY294002. The results indicated that LY294002 pretreatment appeared to alleviate the suppressive impact of APi on p-NF-κB p65 (**Fig 6A**). It is also worth noting that APi appeared to have an inhibitory activity on TNF-α, IL-1β and IL-6 levels. However, LY294002 treatment may have attenuated the suppressive activity of APi on inflammatory mediators expression (**Fig 6B–6D**). These findings indicated that APi may suppress the development of inflammatory mediators, and exert neuroprotective effects through the PI3K/AKT/NF-κB signaling axis.

## Discussion

PD imposes a heavy burden on individuals and economies around the world, and research into the pathogenesis and molecular mechanisms of PD has made great progress over the years. However, single-gene or single-channel pharmaceuticals have not achieved satisfactory results in clinical trials for PD [33, 37, 38]. This may be because PD pathogenesis involves complex genetic, environmental, and other factors that are difficult to pinpoint [39]. In addition, the pathology of PD may involve multiple neurotransmitters and multiple pathways, affecting both the central nervous system and the periphery [40]. Therefore, multi-target, multi-pathway and multi-component drugs are a growing trend in PD drug development [33, 41]. Coincidentally, TCM is rich in primary and secondary metabolites that can improve health problems in this way. This study employed network pharmacology methods to find six active ingredients of Lingxiaohua (apigenin, beta-sitosterol, sitogluside, oleanolic acid, mandenol and linolenic acid ethyl ester) and ten core targets that are highly correlated with PD treatment (TNF, AKT1, INS, TP53, CASP3, JUN, BCL2, MMP9, FOS and HIF1A). The main pathways involved were shown to be PI3K/AKT, Apoptosis and TNF pathways. Multiple components of Lingxiaohua may affect inflammatory and apoptotic events in the pathogenesis of PD by modulating inflammation and apoptosis-related targets and pathways. This research direction offers a potential avenue for the verification of the efficacy of Lingxiaohua in the therapeutic of PD.

Through Ingredient-Target-Pathway network, APi was identified as the main active ingredient of Lingxiaohua. APi is a flavonoid natural compound found in natural plants such as Lingxiaohua. APi can cross the blood-brain barrier [42], and was found to attenuate the expression of IL-6, TNF-α, and IL-1β in the brain tissue of MPTP-induced PD mice [43]. APi has the ability to attenuate functional and structural changes in LPS-induced PD rats via NF-κB/Nrf2 pathway [44]. APi may potentially function as a neuroprotective factor in rotenone-derived PD rat models by inhibiting neuroinflammation as well as oxidative stress-mediated apoptosis [45]. Neuro- inflammation represents a pivotal pathomechanism of PD, and there is evidence of a relatively low prevalence of idiopathic PD in patients taking long-term anti-inflammatory medications [46, 47]. Moreover, it is postulated that inflammatory processes within the gut may influence the pathogenesis of PD through the modulation of alterations in the gut-brain axis [48]. Network pharmacology posits that the TNF and NF-κB signaling pathway represents a pivotal pathway in Lingxiaohua for PD. TNF pathway, which is initiated by the pro-inflammatory cytokine TNF. TNF-α induces a plethora of intracellular responses, including the increase of inflammatory medium, cell proliferation, and cell death [49]. TNF-α exerts its biological benefits primarily by engaging to 2 tumor necrosis factor receptors (TNFR), including TNFR1 and TNFR2. Upon binding to the receptor, TNF-α is able to recruit adaptor molecules, leading the activation of several signaling pathways within the cell, which involve the NF-κB pathway [50]. NF-κB upregulated in Parkinson’s striatum and exerts a vital role in the adjustment of stress responding and inflammatory reactions [51]. Normally, NF-κB consists of three subunits (p50, p65 and IκBα) aggregated in the cytoplasm and is not biologically active. Activating the NF-κB pathway can lead to phosphorylating IκBα. This phosphorylation in turn triggers the ubiquitination of IκBα. After IκBα degradation, free NF-κB (p50-p65) is transported to nucleus where it activates inflammatory mediators such as IL-6, TNF-α, and IL-1β [52–54]. The preceding studies indicate that NF-κB pathway is noteworthy contributor for the pathogenesis of PD. Our study proved that MPP^+^ induced overexpression of inflammatory mediators. Additionally, it led to nuclear translocation of NF-κB p65 subunit through activation of NF-κB pathway in MES23.5 cells. APi could reduce the release of inflammatory mediators by suppressing NF-κB pathway and inhibit the malignant cycle of neuroinflammation.

**Fig 7:**
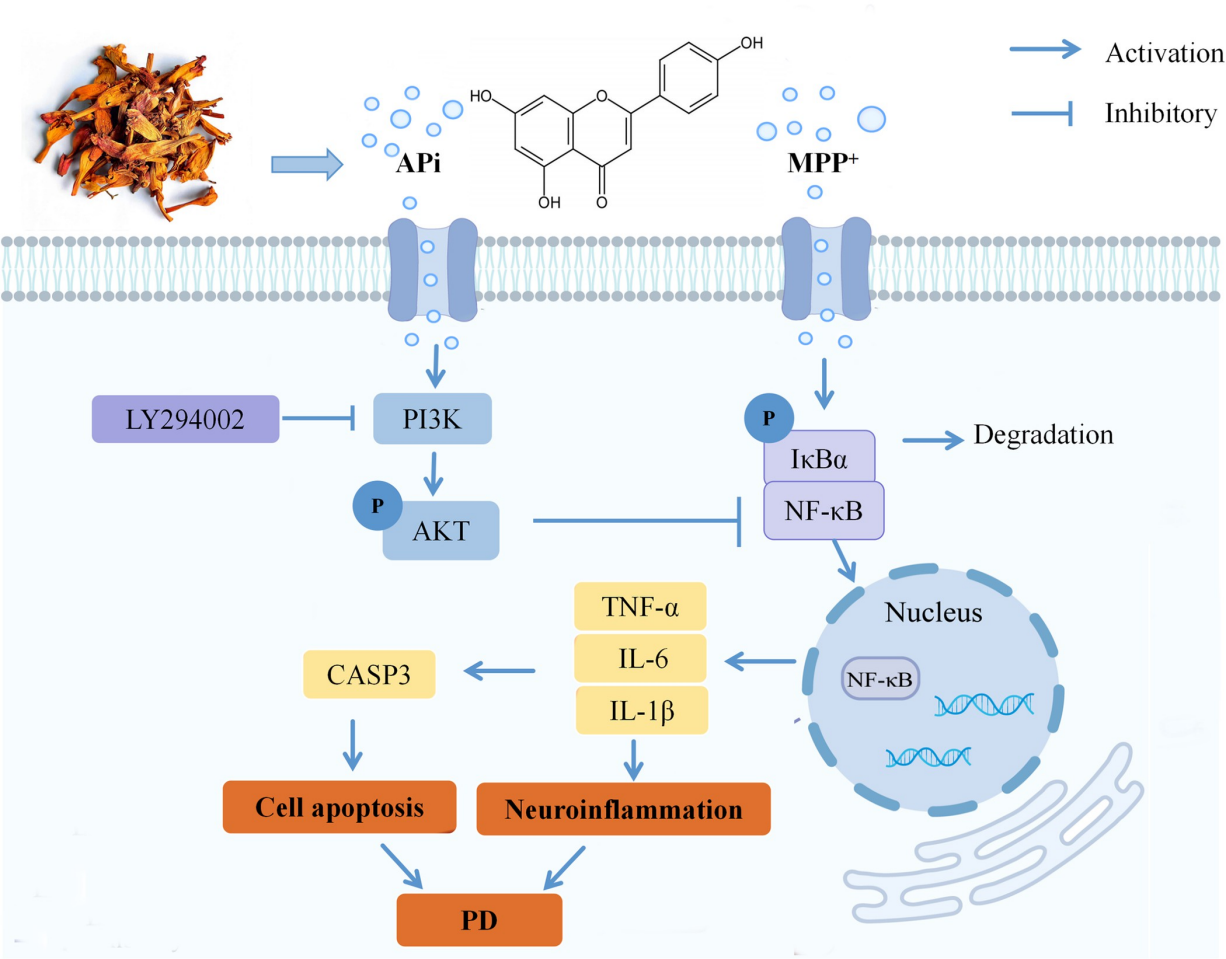
APi as the active ingredient of Lingxiaohua against PD by inhibiting the PI3K/AKT/NF-κB pathway.

The PI3K/AKT and Apoptosis pathways were the key pathways by KEGG enrichment analysis. TNF, AKT1 and CASP3 are the key targets by PPI network construction. In the TNF pathway described above, TNFR1 can activate the downstream protease CASP3 by binding to Fas-related death domain proteins, triggering an apoptotic process that is dependent on the cascade reaction of Caspase [33]. In cellular experiments, we also confirmed that APi inhibited CASP3 activity in cells and its mediated apoptosis. The PI3K/AKT pathway is engaged in modulation of several biological activities, such as autophagy and synaptic plasticity, and is essential in central nervous system (CNS) [55]. The available evidence is mounting to suggest that certain natural products may have the capacity to exert neuroprotective effects through their ability to activate the PI3K/AKT pathway [19]. AKT1 is a pivotal element within the PI3K/AKT signalling cascade, with the NF-κB pathway representing a downstream effector of AKT1 [19, 56]. It has been demonstrated in multiple studies that PI3K/AKT pathway seems a pivotal mediator of anti-inflammatory effects, leading to inhibition of nuclear translocation of NF-κB [3, 57]. Through I-T-P network, APi was identified as the main active ingredient of Lingxiaohua. The 5 most prominent targets in the network were AKT1, BCL2, RELA (NF-κB p65), CASP3 and NFKBIA. As previously described, APi can inhibit MPP^+^-induced NF-κB p65 nuclear translocation to reduce the release of inflammatory mediators and further inhibit CASP3-mediated apoptosis. This further validated the close association of APi with NF-κB p65 and CASP3, as well as the reliability of network pharmacology. Since AKT1 acts as an upstream effector of NF-κB p65, it is reasonable to assume that the anti-inflammatory activity of APi is likely to be mediated through the PI3K/AKT pathway. Our results suggest that APi, as the active ingredient of Lingxiaohua, can inhibit MPP^+^-induced NF-κB p65 phosphorylation and inflammatory mediators expression in MES23.5 cells by activating the PI3K/AKT pathway. The aforementioned outcomes indicate that APi may suppress inflammatory factors levels via PI3K/AKT/NF-κB pathway, thereby exerting neuroprotective effects (**Fig 7**). Although herbs and their natural compounds have a favorable safety and tolerability profile in protecting against CNS damage [19, 58], but there may be risks when used in combination with prescription drugs. Hepatotoxicity, nephrotoxicity, blood pressure, blood glucose and other problems have been reported with some drugs when used in combination with herbs [59]. However, the wide availability, relative safety and accessibility of natural products make this research extremely interesting.

## Conclusion

This study represents the first demonstration that APi, as the main bioactive ingredient of Lingxiaohua, may block the NF-κB pathway via triggering the PI3K/AKT pathway, effectively blocking inflammation-mediated damage to MES23.5 dopaminergic neurons. Results from cellular experiments also confirm the reliability of network pharmacology. In addition, the present study has some limitations and needs to be validated by further studies in animal models of PD. Although no clinical reports are available regarding nutritional supplements used to treat PD, preclinical and clinical studies continue to show some value in diet as a preventive measure. This provides theoretical support for the potential use of Lingxiaohua as a drug or healthy dietary therapy to combat PD.

## Supporting information

S1 Table Active ingredients of Lingxiaohua. (DOCX)

S1 File Original western blot gel image data. (PDF)

S2 File Raw data of all figure results. (XLSX)
