# Supplementary material for: Network pharmacology combined with experimental validation show that apigenin as the active ingredient of Campsis grandiflora flower against Parkinson’s disease by inhibiting the PI3K/AKT/NF-κB pathway
Source: PLoS One. 2024 Oct 9;19(10):e0311824. doi: 10.1371/journal.pone.0311824 (PMC11463827; doi:10.1371/journal.pone.0311824)
Supplement: S1 Table — (DOCX) [file pone.0311824.s001.docx]

S1 Table Active ingredients of Lingxiaohua

| Number | MOL ID | Molecule Name | InCHI key | Molecular formula | Structure formula | MW (g/mol) | OB (%) | DL |
| --- | --- | --- | --- | --- | --- | --- | --- | --- |
| 1 | MOL012358 | 2α,3α-dihydroxyurs-12-en-28-oic aid | HFGSQOYIOKBQOW-BCWNICLQSA-N | C_30_H_48_O_4_ | 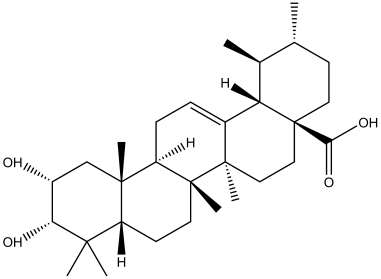 | 472.78 | 28.84 | 0.74 |
| 2 | MOL012362 | Capsanthin | VYIRVAXUEZSDNC-RDJLEWNRSA-N | C_40_H_56_O_3_ | 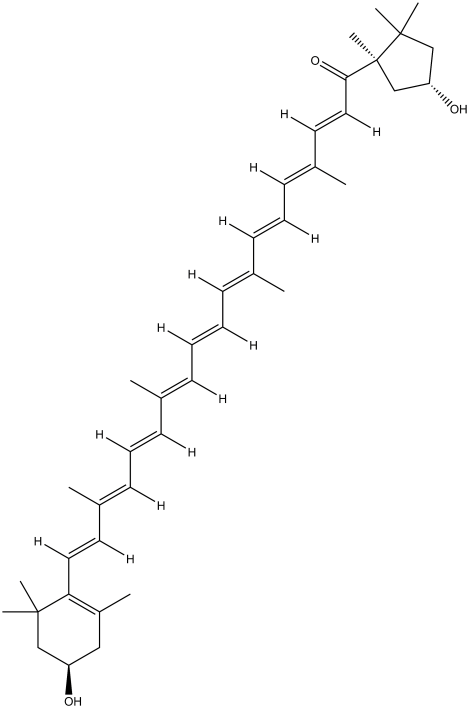 | 584.96 | 47.52 | 0.51 |
| 3 | MOL001494 | Mandenol | FMMOOAYVCKXGMF-MURFETPASA-N | C_20_H_36_O_2_ | 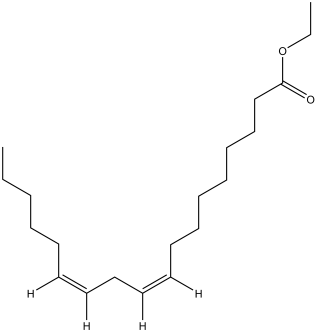 | 308.56 | 42 | 0.19 |
| 4 | MOL000263 | oleanolic acid | MIJYXULNPSFWEK-GTOFXWBISA-N | C_30_H_48_O_3_ | 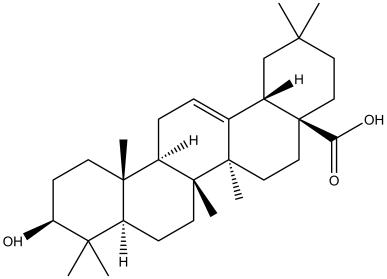 | 456.78 | 29.02 | 0.76 |
| 5 | MOL000357 | Sitogluside | NPJICTMALKLTFW-OFUAXYCQSA-N | C_35_H_60_O_6_ | 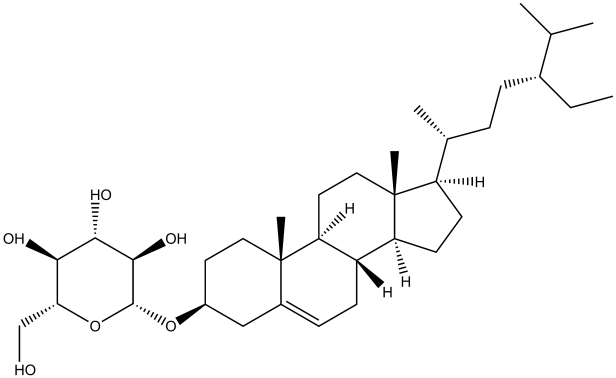 | 576.95 | 20.63 | 0.62 |
| 6 | MOL000358 | beta-sitosterol | KZJWDPNRJALLNS-VJSFXXLFSA-N | C_29_H_50_O | 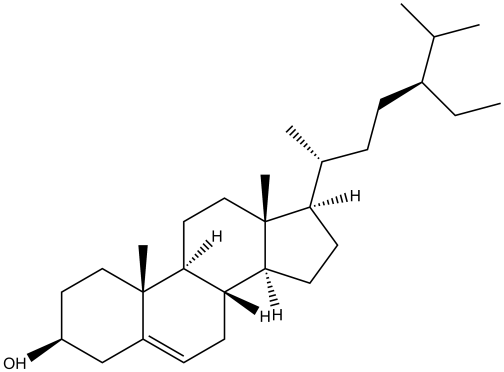 | 414.79 | 36.91 | 0.75 |
| 7 | MOL000551 | Hederagenol | PGOYMURMZNDHNS-MYPRUECHSA-N | C_30_H_48_O_4_ | 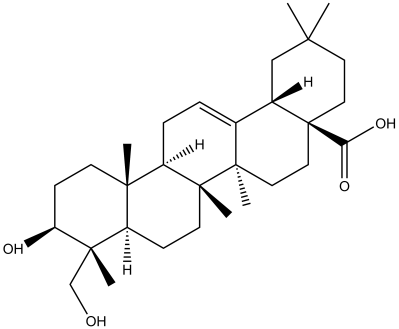 | 472.78 | 22.42 | 0.74 |
| 8 | MOL007179 | Linolenic acid ethyl ester | JYYFMIOPGOFNPK-AGRJPVHOSA-N | C_20_H_34_O_2_ | 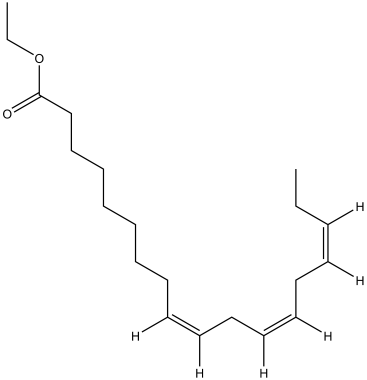 | 306.54 | 46.1 | 0.2 |
| 9 | MOL007254 | Arjunolic acid | RWNHLTKFBKYDOJ-DDHMHSPCSA-N | C_30_H_48_O_5_ | 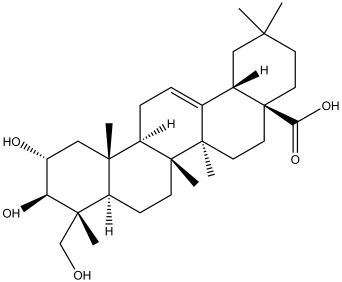 | 488.78 | 23.22 | 0.72 |
| 10 | MOL000008 | apigenin | KZNIFHPLKGYRTM-UHFFFAOYSA-N | C_15_H_10_O_5_ | 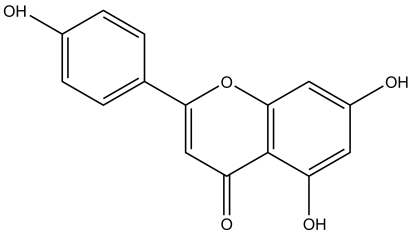 | 270.25 | 23.06 | 0.21 |

Notes: MW, Molecular Weight; OB, Oral Availability; DL: Drug-likeness.
