# Supplementary material for: Network pharmacology combined with experimental validation show that apigenin as the active ingredient of Campsis grandiflora flower against Parkinson’s disease by inhibiting the PI3K/AKT/NF-κB pathway
Source: PLoS One. 2024 Oct 9;19(10):e0311824. doi: 10.1371/journal.pone.0311824 (PMC11463827; doi:10.1371/journal.pone.0311824)

Fig 3B Original western blot for three repeats

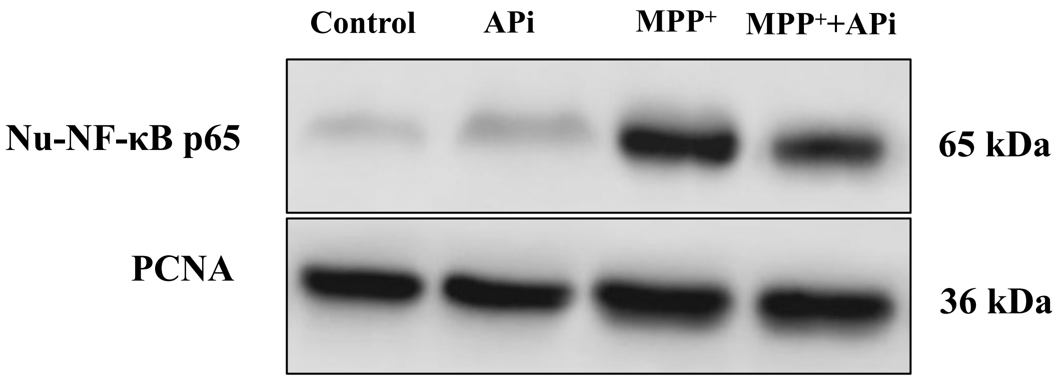

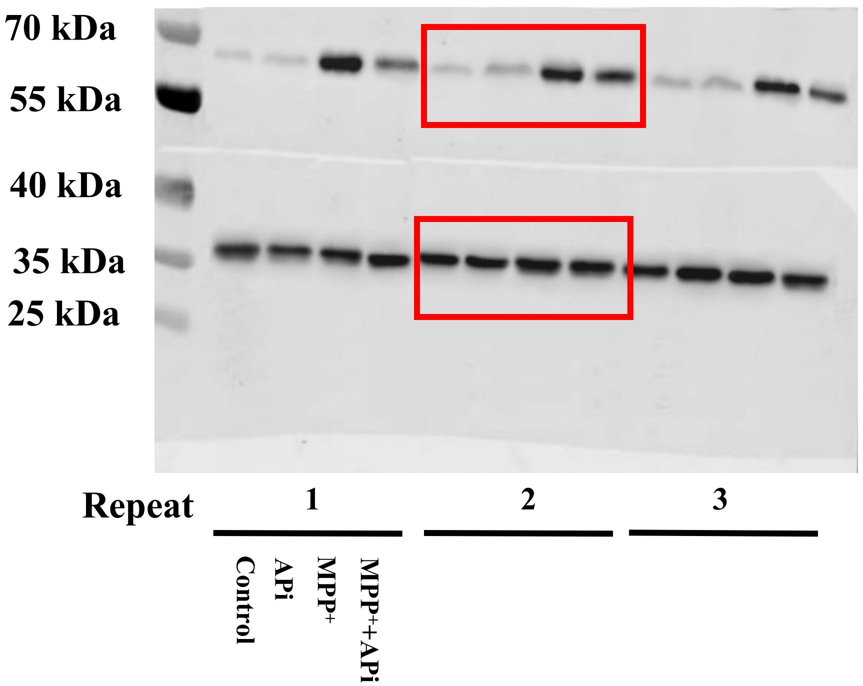

Fig 5A Original western blot for three repeats

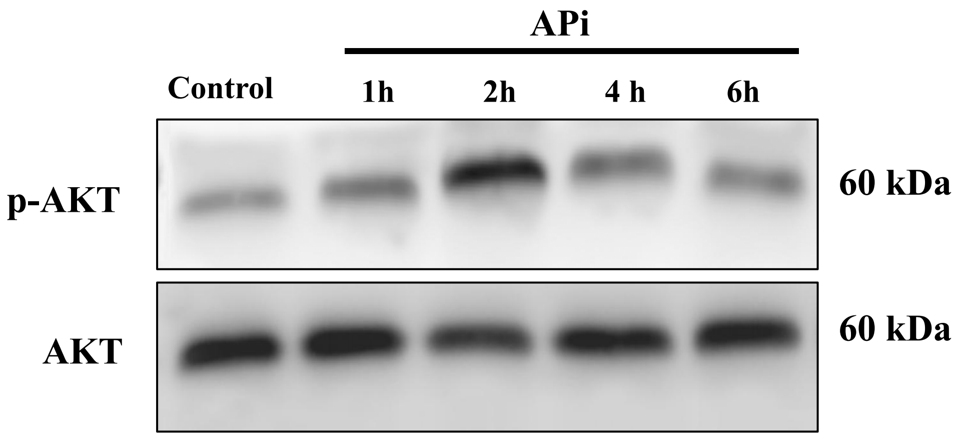

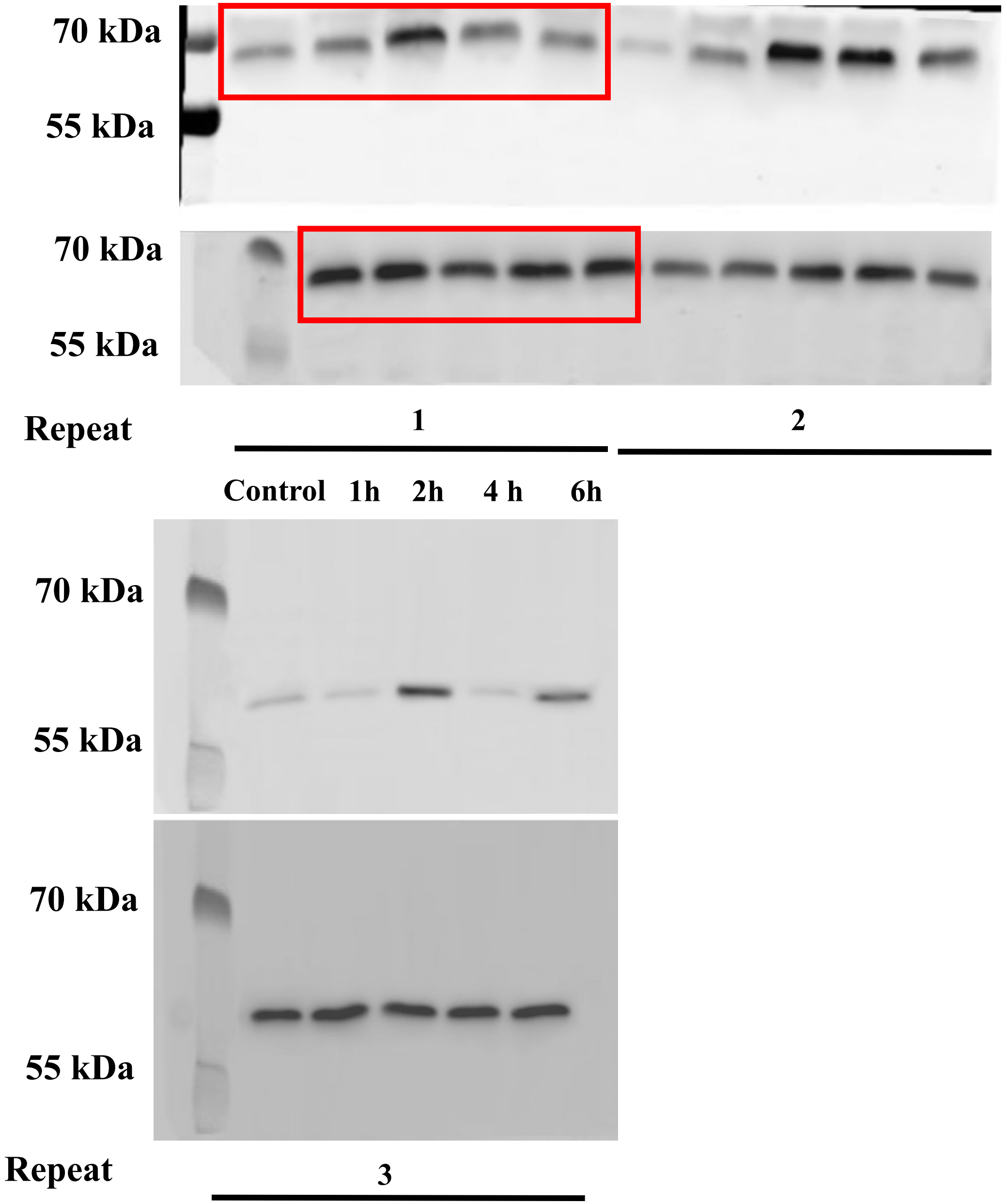

Fig 5B Original western blot for three repeats

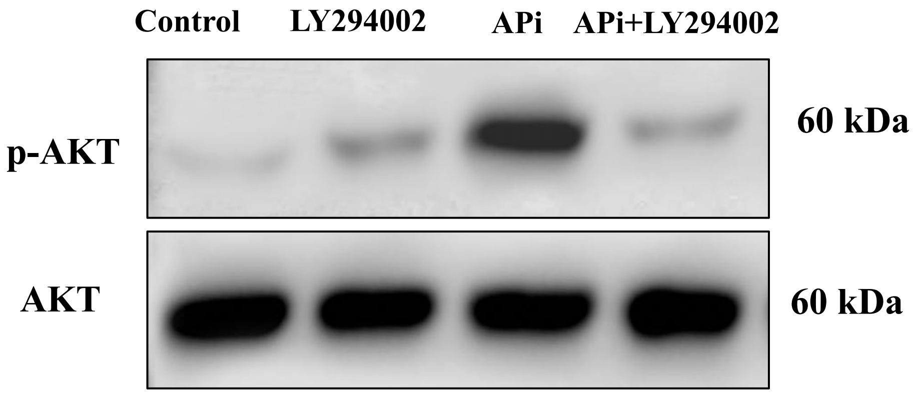

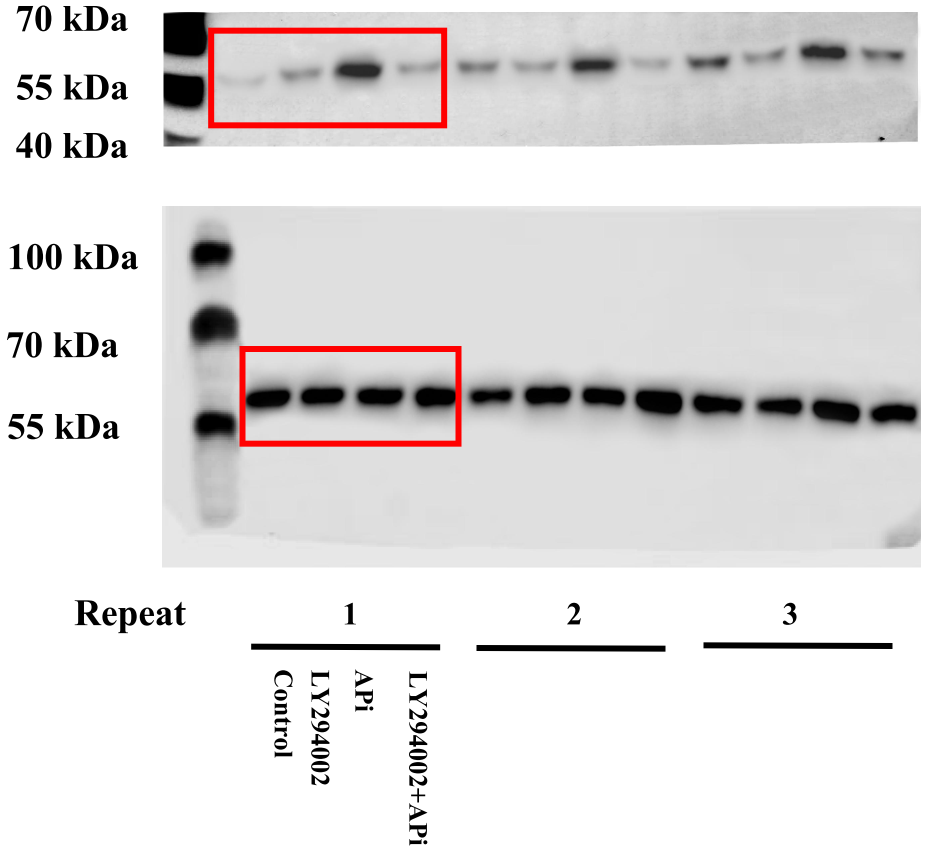

Fig 6A Original western blot for three repeats

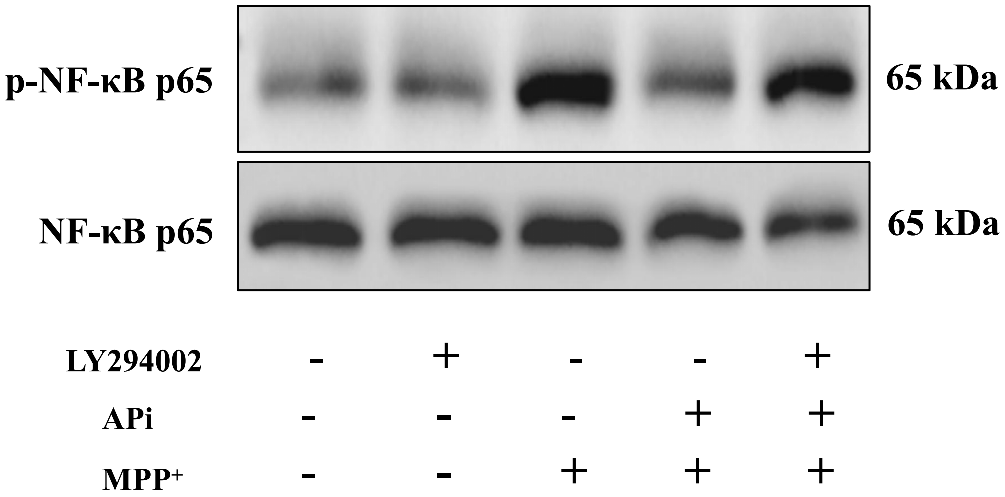

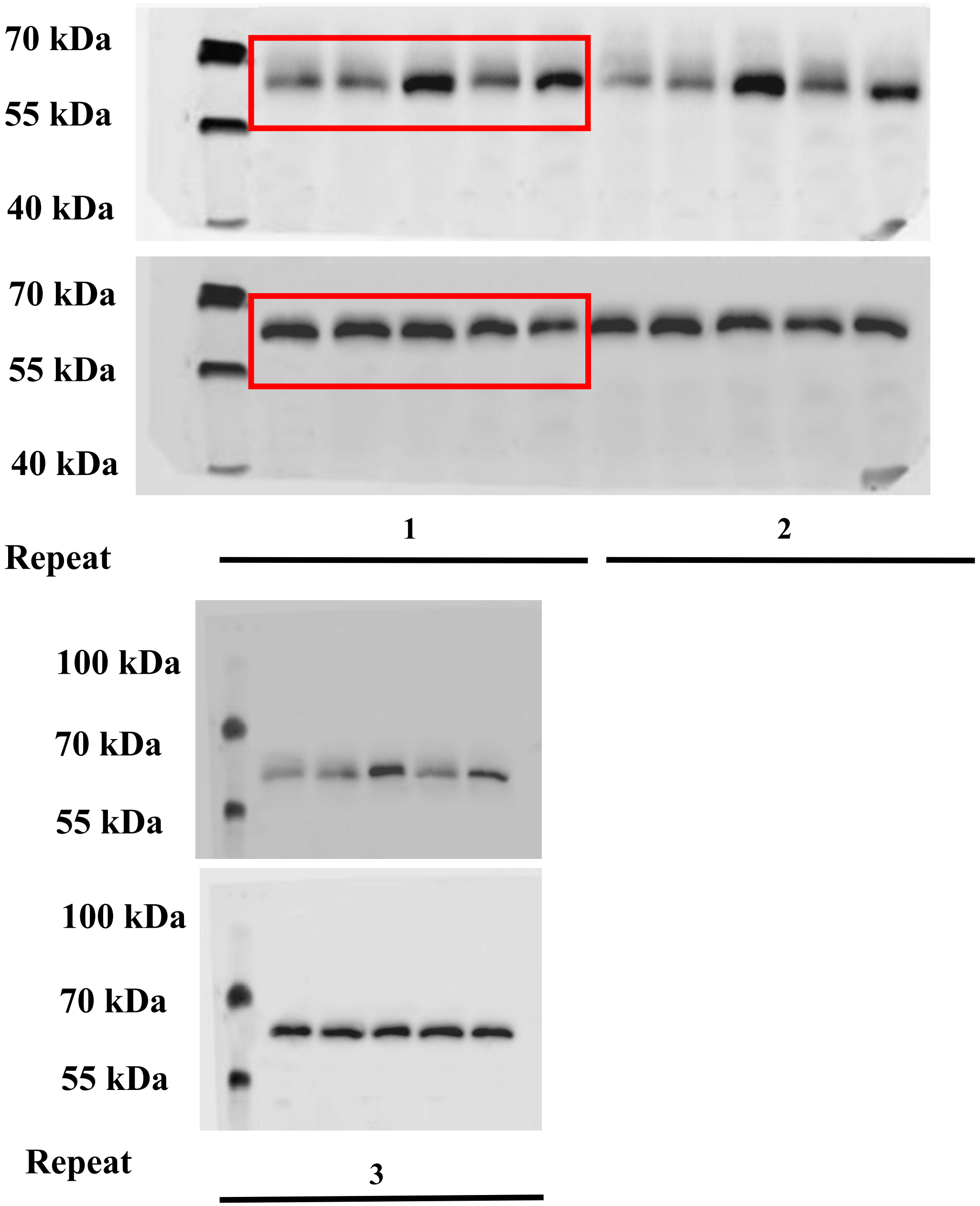

Supplement: S1 File — (PDF) [file pone.0311824.s002.pdf]
